# Impact of Metabolism on Immune Responses

**DOI:** 10.1155/2018/5069316

**Published:** 2018-07-26

**Authors:** Abdallah Elkhal, Hector Rodriguez Cetina Biefer, Miguel A. de la Fuente

**Affiliations:** ^1^Division of Transplant Surgery and Transplantation Surgery Research Laboratory, Brigham and Women's Hospital, Harvard Medical School, Boston, MA, USA; ^2^Division of Cardiovascular Surgery, University Hospital Zurich, Zurich, Switzerland; ^3^Instituto de Biología y Genética Molecular, University of Valladolid, Valladolid, Spain

Metabolic processes have been long seen as means to generate energy in a live organism. However, recent discoveries on the field of immunology and metabolism have shown an increasing interrelation between these processes. The field of immunometabolism is gaining momentum and presents a huge impact for the clinical practice. Of particular attention is the chronic inflammation state in obesity, as well as asthma to mention a few.

This special issue offers a selected and articulated overview of the examined topic. For this issue, we have included an interesting mix of reviews and original research articles, which underscore the relevance of metabolism in immune responses as observed in the listing below:

Y. Yuan et al. describe the immune regulators of lipid metabolism in obesity as well as the interplay between obesity and asthma. They further propose targeted therapies for direct and indirect immune regulators of lipid metabolism, which might help in the treatment of obesity-related asthma.

In the field of cardiovascular diseases, W. Zhou and M. Zhao underscored the importance of the Hippo-signaling pathway, known to regulate multiple organ development and diseases, and explored novel therapeutic approaches in this field.

In the research article by M. H. Lee et al., they explore the effects of sex and estrogens on the IFN signature of marrow-derived DCs in mice. Their findings show that estrogen enhances marrow-derived DC activation through IFN-dependent and -independent pathways, among other interesting findings, suggesting that immunometabolism plays a significant role in sex-biased diseases.

In his review, J. Kim explores molecular mechanisms responsible for molecular reprogramming in macrophages and T cells, further providing recent updates on functional modulation of immune cells by metabolic changes in the microenvironment.

H. Li et al. were able to show how intermittently high glucose potentiates activation and inflammatory responses through Toll-like receptor 4 and Th1 cells, suggesting a more detrimental role of glucose in a diabetic patient than generally assumed, in particular in diabetes-related vascular diseases.

H. Zhong et al. describe the role of obesity-induced metabolic dysfunction and its correlation to chronic inflammation. Furthermore, they analyze the role of microRNAs in obesity and suggest possible new targets in this field.

J. L. Grün et al. were able to show the plasticity of monocytes in response to metabolic syndrome risk factors such as high-density lipoprotein in human cells. These findings support the notion that metabolic dysfunction has a pivotal component in the systemic inflammatory response observed in cardiovascular diseases.

A. Mishra focuses on the metabolic processes of dendritic cells involved in the activation and differentiation of dendritic cells with special interest in the implications to control airway inflammation and adaptive immunity in asthma.

